# Modeling habitat suitability of *Dorema ammoniacum* D Don. in the rangelands of central Iran

**DOI:** 10.1038/s41598-024-61073-8

**Published:** 2024-07-13

**Authors:** Mostafa Zare, Mehdi Moameri, Ardavan Ghorbani, Hossein Piri Sahragard, Raoof Mostafazadeh, Farid Dadjou, Asim Biswas

**Affiliations:** 1 Faculty of Agriculture and Natural Resources, University of Mohaghegh Ardabili, Ardabil, Iran; 2 Faculty of Agriculture and Natural Resources, University of Mohaghegh Ardabili, Daneshgah Street, Ardabil, 56199 13131 Iran; 3 Department of Rangeland and Watershed, Water and Soil Faculty, University of Zabol, Zabol, Iran; 4 School of Environmental Sciences, University of Guelph, Guelph, Canada

**Keywords:** Habitat suitability, Prediction models, Environmental variable, Ecology, Ecosystem ecology

## Abstract

The purpose of this study was to evaluate the predictive accuracy of habitat suitability models, identifying the potential distribution range of *Dorema ammoniacum*, and its habitat requirements in the rangelands of Yazd province, central Iran. Bafgh, Mehriz and Nadoushan, were three habitats that were identified, and sampling was conducted in each habitat using a random-systematic method. A set of 10 plots were established (at equal distances) along 350 m long 18 transects. Soil samples (two depths: 0–30 and 30–60 cm from 36 profiles) were collected and measured in the laboratory. Elevation, slope, and aspect maps were derived, and climate information was collected from nearby meteorological stations. The habitat prediction of the species was modeled using Logistic Regression (LR), Maximum Entropy (MaxEnt), and Artificial Neural Network (ANN). The Kappa coefficient and the area under the curve (AUC) were calculated to assess the accuracy of the forecasted maps. The LR model for habitat prediction of the studied species in Mehriz (K = 0.67) and Nadoushan (K = 0.56) habitats were identified as good. The MaxEnt model predicted the habitat distribution for the selected species in Bafgh and Mehriz habitats as excellent (K = 0.89, AUC = 0.76, K = 0.89, AUC = 0.98), and in the Nadoushan habitat as very good (K = 0.78, AUC = 0.85). However, the ANN model predicted Bafgh and Nadoushan habitats as excellent and Mehriz habitat as very good (K = 0.87, K = 0.90, and K = 0.63, respectively). In general, in order to protect species *D. ammoniacum*, the development of its habitats in other areas of Yazd province and the habitats under study in conservation programs should be given priority.

## Introduction

Global biodiversity faces significant threats from various sources, including land use changes, climate change, habitat fragmentation, invasive species expansion, and ecosystem misuse^1^. Particularly vulnerable are threatened and endemic species, characterized by limited geographic ranges, specialized habitats, small populations, and low reproductive rates, making them more susceptible to extinction^2–4^. Despite their vulnerability, these species play crucial roles in ecosystem functioning, contributing to biodiversity maintenance and resilience against invasions, thus underscoring the urgency of their conservation^5,6^. Conservation efforts for these species require comprehensive understanding of their geographic distributions and identification of suitable reintroduction areas, yet data limitations persist due to challenges in data collection, often constrained by accessibility and seasonal constraints^7–10^. Successful reintroduction programs hinge on accurate knowledge of suitable habitats, yet time constraints have limited assessments to only a fraction of species^11,12^.

The distribution of plant species within an area is influenced by a complex interplay of environmental factors and ecological requirements unique to each species, reflecting their varying tolerances to environmental conditions^13,14^. Understanding the impact of these factors is crucial for generating species habitat maps, enabling informed decision-making for sustainable planning and habitat management^15–17^. Predictive mapping of vegetation distribution relies on spatial data encompassing species presence or absence, abundance, and precise environmental variable maps, facilitating the estimation of optimal conditions for species survival^18^.

There are several modeling methods with variable capabilities and limitations for predicting the distribution of plant species, such as logistic regression (LR)^19^, maximum entropy (MaxEnt)^4^, and artificial neural network (ANN)^20^. Given the divergent prediction performances of these methods, comparative studies are essential to select the most accurate approach and quantify prediction errors^21,22^. Evaluation of LR, MaxEnt, and ANN methods in predicting plant species distribution in the rangelands of Hoze Soltan, Qom province, Iran, revealed that the artificial neural network method exhibited the highest prediction accuracy, followed by MaxEnt and LR^23^. However, no single method can be universally deemed optimal for all species. In a separate study, the MaxEnt model and fuzzy logic were utilized to delineate habitat distribution and conservation strategies for endangered species, including *Coronus officinalis* Sieb, in China^24^.

In another study, the logistic regression (LR) model was employed to ascertain the factors influencing the distribution of *Muscari latifolium* J. Kirk. in the mountains of western Anatolia, Turkey^25^. This analysis revealed five dominant variables, which collectively explained 60% of the main factors determining species distribution, with prediction performance assessed using the area under the curve (AUC). Additionally, the MaxEnt model was utilized to forecast the current and future cultivation areas of *Carathamus tictorius* L. in China, considering the impacts of climate change, yielding AUC values exceeding 0.97 for all models^26^. Furthermore, a comparison between LR and MaxEnt models was conducted to delineate the distribution of plant species in Taftan, southeastern Iran^18^.

It was determined that both LR and MaxEnt models exhibited comparable efficacy in modeling the distribution of plant species with limited ecological niches; however, the MaxEnt model demonstrated superior performance, particularly in predicting the distribution of such species, leveraging a smaller dataset of presence/absence records. In a separate investigation, the LR model was employed to delineate the spatial distribution of the invasive species *Eragrostis curvula* (Schrad.) Nees in New Jersey (USA)^27^. The successful prediction probability for the presence of this species was notably high, reaching 82.35%, with an overall prediction accuracy of 80.88%^28^. Additionally, the MaxEnt method was utilized to forecast the potential distribution of the native rose species, indicating the versatility and effectiveness of this modeling approach in species distribution prediction^28^. The study of the species distribution model highlighted the crucial importance of considering uncertainties linked to future climate change scenarios^29^.

The prediction of Leucanthemum vulgare Lam. distribution in the rangelands of Ardabil province using the LR model revealed a positive correlation between the species' presence and soil temperature and volumetric moisture content, emphasizing the predictive accuracy of this method^30^. Additionally, the spatial distribution of the invasive species *Alternanthera philoxeroides* (Mart.) Griseb. in China was successfully predicted using GIS and the MaxEnt model, showcasing the effectiveness of predictive modeling in invasive species management^31^. Furthermore, the distribution of *Prangos uloptera* DC. in the southern rangelands of Ardabil province, Iran, was assessed using LR and MaxEnt models, with MaxEnt identifying the most influential factor for species spread, albeit with lower accuracy compared to LR^32^.

The genus Dorema (Apiaceae) comprises seven species within the Iranian flora, with *D. ammonicum* D. Don, D. *aucheri* Boiss, and D. *glabrum* Fisch. C.A. Mey being endemic to the region^33^. *D. ammoniacum*, specifically, exhibits a unique global geographical distribution, being native exclusively to Iran and thriving in desert regions with lime soil. Its natural habitat spans provinces such as Yazd, Sistan-va-Baluchestan, Isfahan, Semnan, Fars, Kerman, and Khorasan^34,35^. *Dorema ammoniacum*, a perennial monocarpic herbaceous plant found in the desert regions of Iran, holds significant medicinal, industrial, and forage value, often serving as an indicator species within its habitat and dominating or coexisting within plant communities^36^. Renowned for its medicinal properties, the gum resin extracted from this plant has been utilized in Iranian traditional medicine for treating anthelmintic and gastrointestinal disorders, while also finding application in the pharmaceutical industry^37^. Studies have highlighted its antibacterial and vasodilatory effects, with its extract even employed as an anticonvulsant in Greek folk medicine^38^. Despite its importance, factors such as improper exploitation and habitat conversion to agricultural lands have placed *D. ammoniacum* under the threat of extinction. Classified as Vulnerable (VU) by the International Union for Conservation of Nature (IUCN), there remains a scarcity of research examining the factors influencing its distribution and suitable growth areas.

Therefore, the research aimed to achieve two primary objectives: (1) identifying the key environmental factors influencing the distribution of *D. ammoniacum*, and (2) forecasting its potential distribution based on predetermined environmental parameters through LR, MaxEnt, and ANN models. The ultimate goal is to pinpoint areas with heightened suitability for the species' establishment, enabling the integration of these findings into development programs and habitat restoration initiatives within Yazd province.

## Materials and methods

### Study area

**Figure 1:**
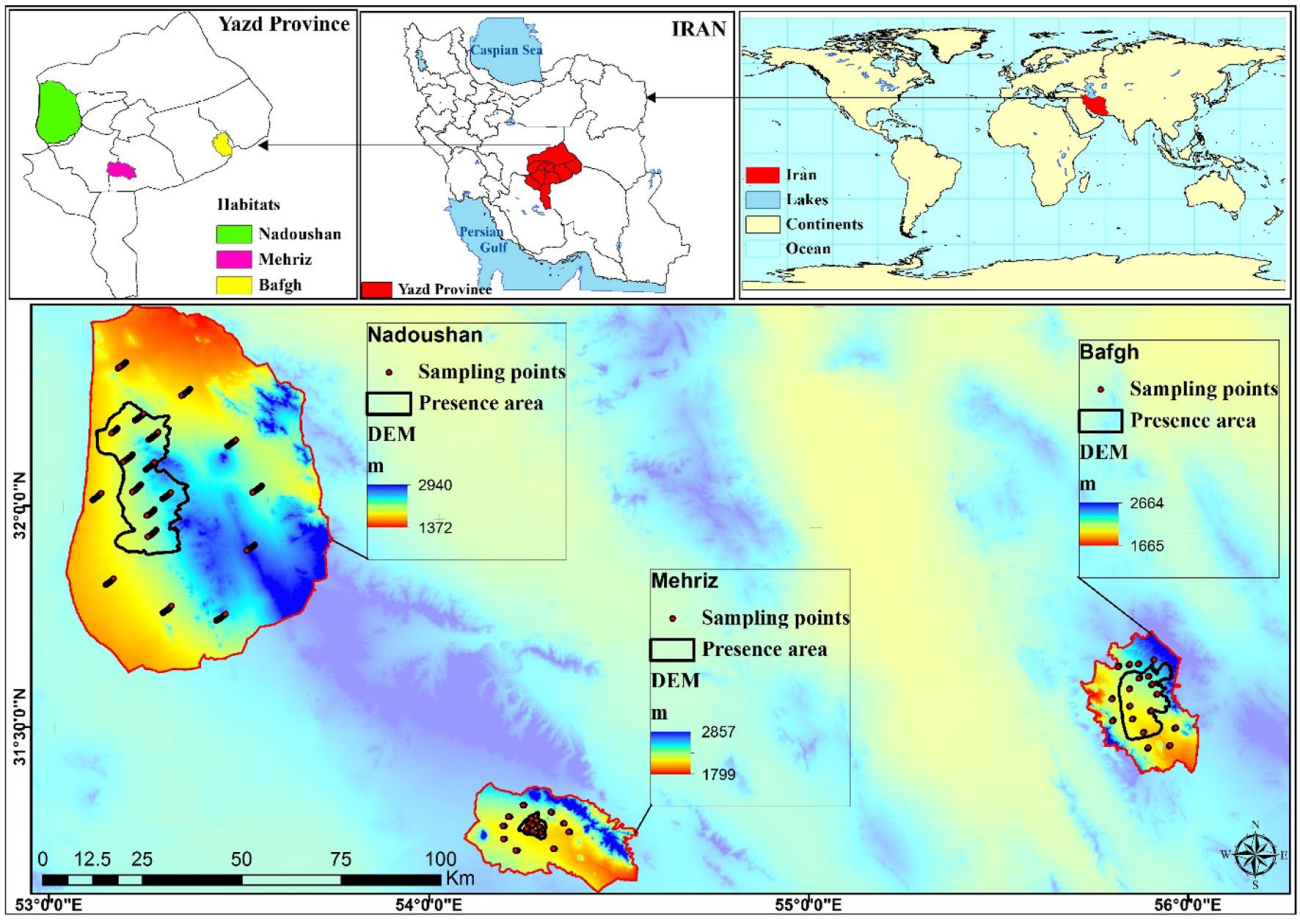
Location of *D. ammoniacum* in Yazd Province and Iran (The processing and creation of maps were conducted by the researchers using ArcGIS version 10.1^39^.

*D. ammoniacum* has three major habitats in Yazd Province, Iran, and is distributed in Nadoushan, Bafgh and Mehriz regions (Fig. 1). These maps of studied areas were generated by the researchers using ArcGIS version 10.1^39^. Furthermore, the key specifications of the study regions are elucidated in the subsequent sections. (i) Gazestan Bafgh is in the southwest of Bafgh county (55° 42' to 56° 2' E and 31° 26' to 31° 47' N). The altitude range is 1800–2600 masl, and the average annual precipitation is 150 mm per year. The average annual temperature is 15.5 °C. The soil texture dominating this area is sandy and loamy. The composition of the livestock herd consists of approximately 70% goats and 30% sheep. (ii) Aliabad Ghelgazi Mehriz is in the west of Mehriz county with a geographical range of 54° 5' to 54° 34' E and 31° 10' to 31° 24' N. The altitude range is 1800–2800 masl. The average annual precipitation is 140 mm per year, and the average annual temperature is 13.5 °C. The composition of the livestock herd consists of approximately 70% goats and 30% sheep. (iii) Nadoushan is in the west of Meybod county with a geographical range of 53° 1' to 53° 45' E and 31° 28' to 31° 38' N. This area is primarily plain with an altitude range of 1370–2900 m above sea level (masl). According to the 22-year statistics of meteorological stations in the study areas, the average annual precipitation is 110 mm, and the average annual temperature is 18.5 °C. Soil texture is sandy and sandy-loamy. According to field observations and surveys conducted with local residents, the composition of the livestock herd consists of approximately 75% goats and 25% sheep. A study of vegetation mapping showed that there are 13 plant types in the habitat area, which is mainly dominated by *Artemisia sieberi* Besser^40^.

Climatic data originated from the Iran Meteorological Organization and were mapped based on station locations. The main characteristics of meteorological stations and climatic variables, based on the averaged values over 22 years of data in the study area's habitats, are detailed in Table 1.

**Table 1:** Characteristics of meteorological stations and climatic variables in the habitats of the study area.

Habitats	Meteorological station	Elevation amsl (m)	Latitude	Longitude	Mean annual precipitation (mm)	Mean annual temperature (°C)
Bafgh	Koushk	1986	55° 47 '	31° 44 '	150	15.5
	Ghotroum	1526	55° 48 '	31° 23 '		
	Bajgan	2065	55° 48 '	31° 45 '		
	Bahadoran	1480	54° 56 '	31° 19 '		
	Bafgh	898	55° 26 '	31° 36 '		
Mehriz	Nir	2450	54° 09 '	31° 48 '	140	13.5
	Mehriz	1520	54° 48 '	31° 57 '		
	Tangchenar	2182	54° 21 '	31° 24 '		
	Arnan	2015	54° 10 '	31° 19 '		
	Aliabad	1915	54° 16 '	31° 17 '		
	Gerdkouh	1457	54° 47 '	31° 30 '		
Nadoushan	Nadoushan	1950	53° 33 '	32° 01 '	110	18.5
	Meibod	1109	54° 01 '	32° 23 '		
	Eghda	1138	53° 37 '	32° 26 '		
	Ardakan	1104	54° 01 '	32° 19 '		
	Sourak	2270	53° 25 '	32° 09 '		
	Mazrae-no Eghda	1370	53° 29 '	32° 24 '		
	Shamsabad Eghda	1147	53° 40 '	32° 26 '		
	Zarjoua	1800	53° 20 '	32° 20 '		

### Data collection

Habitat identification within each region involved a comprehensive approach, combining landform and geological maps, field surveys, and observational data to delineate sampling units^30,41–43^. Following this, areas devoid of the target species were randomly chosen near existing presence areas, ensuring similarity in soil composition and ecological conditions. Subsequently, non-presence sites for the studied plant species were selected through a random process. In each of the identified habitats, sampling was performed using a systematic-random method along 18 sampling transects of 350 m. On each transect, 10 plots of 2 m^2^ were established. The length of sampling transects, and quadrat size was determined based on the density and type of vegetation identified in previous studies^30,41–43^. Two soil profile samples were collected along each transect. The depth of the profiles was determined according to the effective rooting depth of the studied species. As the maximum rooting depth was identified as 60 cm on average^30^, two soil samples were collected from 0–30 cm as the surface soil and 30–60 cm as the subsurface soil. A total of 36 soil samples were collected from each habitat. Then, several soil physical and chemical properties were measured in the laboratory, including texture (Bouyoucos hydrometer method), saturation moisture (SP), electrical conductivity (EC) (saturated extraction method), acidic and alkaline (pH) (potentiometric method), organic carbon (OC) and organic matter (OM) Walkely and Black^44^, soluble solutes including Na and K (flame photometer method), Ca, Mg, K, Cl–, CO_3_– and HCO_3_– (titration method) and SO_4_- (spectrophotometer measured in the laboratory)^30,32^. The geographical location of the sampling points was recorded using a hand-held Global Positioning System (GPS). Soil factor maps affecting the habitat of D. ammoniacum were generated through Kriging interpolation using ArcGIS version 10.1^39^. Soil factor coefficients were derived from regression analysis and then integrated into the environmental layers. The desired parameters for each sampling point were then extracted from slope, aspect, and altitude maps by the researchers using ArcGIS version 10.1^39^. Meteorological data (average annual precipitation and temperature) were collected from the meteorological stations from the study region. Total data were organized in Excel according to different habitats, followed by a check for normality using Kolmogorov–Smirnov and Anderson Darling tests. If normalization was necessary, methods such as log, square-root, and Box-Cox transformation were applied; otherwise, non-parametric geostatistical methods like inverse distance weighting (IDW) were utilized. Prior to model application, initial multicollinearity among independent factors was assessed, and factors with VIF > 10 were eliminated^45^. Environmental factor maps were generated using ArcGIS 10.1 software, and Table 2 provides details on the total number of variables, their determination method, and corresponding references.

**Table 2:** Environmental variables in modeling habitat suitability of *Dorema ammoniacum* D Don.

Variable	Methods	Reference
Sampling method	Systematic-random	^30,41–43,46,47^
Number of transects	Based on the density and distribution of vegetation cover	^30,41–43,46,47^
Transect length (m)	Based on the distribution of vegetation cover	^30,41–43,46,47^
Number of plots	Based on the density and distribution of vegetation cover	^30,41–43,46,47^
Plots size (m^2^)	Minimal area	^30,41–43,46,47^
Depth of soil profile (cm)	Based on the rooting depth	^41–43^
Soil texture	Bouyoucos hydrometer method	^48^
Saturation percentage (SP) (%)	Weight method	^49^
Electrical conductivity (EC) (dS/m)	Soil–water saturated extraction (Using EC meter)	^50^
pH	Soil–water saturated extraction (Using pH meter)	^50^
Total nitrogen (%)	Kjeldahl method	^51^
Organic carbon (OC) and organic matter (OM) (%)	Walkely and Black method	^52^
Soluble Na (ppm), Soluble K (ppm)	Flame photometer method in 1:2 soil: water extract	^53^
Ca, Mg, HCO_3_, Cl, CO_3_, and SO_4_	Titration method with EDTA in 1:2 soil: water extract	^54^
Data normality	Kolmogorov–Smirnov and Anderson Darling tests log, square-root, and Box-Cox transformation methods Inverse distance weighting (IDW)	^55–59^
Multi-collinearity	VIF > 10	^45^
Average annual precipitation	Meteorological stations	^60^
Average annual temperature	Meteorological stations	^60^
Slope	GIS	^30,41–43,46^
Aspect	GIS	^30,41–43,46^
Altitude	GIS	^30,41–43,46^

### Data analysis

In this study, three machine learning algorithms were employed to predict plant habitat: Logistic Regression (LR), Maximum Entropy (MaxEnt), and Artificial Neural Networks (ANN). These methods offer diverse approaches to modeling and analyzing habitat distribution, each with its own strengths and applications.

#### Logistic regression (LR) model

Logistic Regression (LR) is a highly valuable method for exploring the relationship between independent variables and a binary response variable, such as the presence or absence of a specific plant species. As a specialized form of multiple regression, LR is particularly adept at analyzing discrete dependent variables. Presence-absence models, like logistic regression, frequently yield ecologically sound relationships, providing valuable insights into species distribution dynamics^61,62^. In this study, the logistic regression model, as represented by Eq. 1, was employed to predict the presence and absence of plant species^63^. In LR model, a dataset comprising 180 points is utilized for modeling. This dataset consists of 90 points indicating species presence and 90 points indicating species absence. Here, plant species served as the dependent variables, while environmental factors acted as the independent variables. Coefficients were assigned to each layer within ArcGIS, facilitating the generation of prediction maps for the studied species across different habitats. These maps provided a continuous probability range from 0 to 1 based on the presence or absence of *D. ammoniacum* species. The logistic regression model was implemented using SPSS_Ver.18_ software.

1 $$Y = Exp\left( {b_{0} + b_{{1}} x_{{1}} + \ldots + b_{{\text{n}}} x_{{\text{n}}} } \right)/{1} + Exp\left( {b_{0} + b_{1} x_{{1}} + \ldots + b_{{\text{n}}} x_{{\text{n}}} } \right),$$

where Y is the probability of occurrence of the species, x are the predictive variables and b are the regression model coefficients.

#### MaxEnt model

The Maxent model, a species distribution model derived from machine learning, is utilized to forecast the potential distribution of species^64,65^. This model, which estimates species distribution based on presence-only data, has demonstrated practical effectiveness^21,32^ and can aid in predicting the detectability of a species^66–68^. Incorporating both continuous and categorical data as well as interactions between variables, this method has been shown to create satisfactory results even with limited sample sizes. By determining the optimal distribution function between species presence points and environmental variables using the maximum entropy principle, the MaxEnt model then extrapolates this function across the study area to generate a distribution map of the target species^21,69^.

In this method, first, the environmental layers are evaluated as the location of training data, and then the probability of occurrence of the studied species in the whole region is selected^32,68^. Data of 90 occurrence points from sampling sites were used for model building. In regards to spatial thinning, it’s important to mention that the selection of sampling sites and plant presence locations has been accurately designed to prevent false replicates and ensure representative soil samples of suitable spatial distributions based on environmental diversity. The rationale behind the research design was to carefully select sampling sites and locations where the plant species is present in order to avoid redundant data points and ensure a diverse representation of environmental factors. This approach was intended to facilitate a spatially balanced distribution of samples of the target species and soil samples, aligning with the research objectives.

Maps of environmental factors built into ASCII template, and MaxEnt 3.3 software applied for modeling the species distribution. In the algorithm, the number of iterations was set to 5000, with a convergence threshold of 0.00001. A random test percentage of 25 was allocated for test data, and the "Random seed" option was checked for replicate run types. Additionally, a subset was utilized for most of the implemented models, with 10,000 background points, a regularization multiplier of 1, and 15 runs in the replicates field. The output grid format was logistic, and the algorithm factors were set to "auto features", with default values chosen for other options^70^. In the process, 25% of the data were separated for model testing (accuracy assessment) and the remaining data were used for training^67^. The receiver operating characteristic curve (ROC) was obtained to test the efficiency of the MaxEnt model. Additionally, the area under the curve (AUC) value was computed for assessing the precision of the model. AUC presents the overall accuracy of the model^68^. The value range for AUC is from 0 to 1^71^. AUC scores above 0.5 indicate the model performs better than random and above 0.75 are considered acceptable for predictive accuracy^72^, with > 0.8 considered good and 0.9 considered excellent^73^. The AUC statistic presented the model’s superiority in recognition among existence and non-existence areas. The statistical value near unity showed a better consistency of the prediction model with the recorded sampling data and reality^74^. We applied the Jackknife test to distinguish the most effective environmental factors^18^. Jackknife of regularized training gain is very important in determining the most important environmental factors affecting species distribution. If this model is implemented separately, it compares the training gain of each variable and then compares it with all environmental variables^10,32^. The probability of species presence ranging from 0 to 1 was estimated^75^.

#### Artificial neural networks (ANN) model

ANN is a species distribution model that predicts the distribution of a species using presence and absence data. ANN is far from the drawbacks of traditional systems and has the ability to learn and generalize using the examples provided during the training phase. This model is a simulation method and one of the most common classifications for pattern resetting^76^. The advantage of the neural network method is to learn directly from the data, without the need to estimate their statistical characteristics^77^. The neural network is able to find the relationship between a set of inputs and outputs to predict any output corresponding to the desired input and is able to estimate any type of nonlinear function^78,79^.

In ANN model, a dataset comprising 180 points is utilized for modeling. This dataset consists of 90 points indicating species presence and 90 points indicating species absence. TerrSet 18.31 software facilitated the implementation of ANN. The concept of ANN was first introduced by Rosenblatt, who pioneered multilayer perceptron networks (MLP)^80^. These networks consist of input, hidden, and output layers, with the number of neurons in each layer determined by the specific problem being addressed. The configuration of hidden layers and their neurons is typically optimized through trial and error to minimize errors^81,82^. The process of utilizing MLP involves creating, training, and validating the network. During network creation, inputs and outputs are defined, and model performance is assessed using a quadratic polynomial equation (Eq. (2))^83^. The training phase involves iteratively adjusting network parameters to minimize the root mean square error (RMSE) using a subset of the data.

2 $$z_{ij} = \, a \, + \, bx_{i} + \, cx_{j} + \, dx_{i} + \, ex_{j} + \, fx_{i} x_{j} ,$$

where x_i_ and x_j_ are any pairs of the independent variables, while z_ij_ is the predicted output from the first layer of neurons in the GMDH algorithm. The described procedure is repeated for a given set of ‘n’ observation of the m independent variables (x_1_, x_2_, > … > , xm). As the number of input variables is m, the total number of combination of each pair of variables x_1_ and x_j_ is m (m − 1)/2.

The processing and creation of spatial change maps resulting from species distribution modeling were conducted by the researchers using ArcGIS version 10.1^39^, employing diverse functionalities such as spatial and 3D analysis, and subsequently presented as spatial maps.

To validate the maps obtained from the model prediction, the presence data that were not analyzed were used as a reference image. Also, in this study, Kappa statistics were used to assess the performance of the models by comparing the probability forecast map with the actual map. To compare the observed and predicted maps, a cut-off point is required to convert continuous probabilities to binary probabilities (presence and absence). In the present study, the presence points predicted in the prediction maps and the presence points in the actual maps were calculated using SPSS_Ver. 18_ software. After determining the optimal threshold, the presence and absence maps of the studied species were classified based on this threshold. Then, the Kappa coefficient was used to evaluate the accommodation between the forecast map and the real map (Eq. (3)). The accuracy of the forecast maps and real maps were tested in TerrSet 18.31 software^15,32^.

3 $$K=\frac{\left(\frac{a+d}{n}\right)-\frac{\left(a+b\right)\left(a+c\right)+(c+d)(d+b)}{{n}^{2}}}{1-\frac{\left(a+b\right)\left(a+c\right)+(c+d)(d+b)}{{n}^{2}}}.$$

### Ethics approval and sampling permissions

All experimental procedures underwent approval from the Review Board of the Department of Range and Watershed Management, Faculty of Agriculture and Natural Resources, University of Mohaghegh Ardabili, Iran. Adherence to applicable guidelines and regulations was strictly observed throughout the study. Notably, no sampling of D. ammoniacum occurred, and there was no harvesting of any part of the plant. Permission for research activities within the study area was granted by the General Department of Natural Resources and Watershed Management of Yazd Province, Iran. Furthermore, the species has previously been identified, and its habitat has been modeled in this research.

## Results

### Logistic regression

In the Nadoushan habitat, soil parameters emerge as the primary influencers of *D. ammoniacum* distribution, as indicated by Eq. (4). Notably, the presence of D. ammoniacum exhibits a positive correlation with certain soil characteristics, including the percentage of saturated moisture (SP), calcium (Ca), and sodium (Na) within the surface layer (0–30 cm), as well as organic carbon (OC) within the subsurface layer (30–60 cm). Conversely, the species presence shows a negative association with nitrogen (N) levels within the surface layer. According to the specified optimal threshold (0.21), the degree of accommodation of the forecast map with the real map is at a good level (0.556) (Table 5).

Soil parameters are also the most important factors in the distribution of this species in the Bafgh habitat (Eq. (5)). The presence of *D. ammoniacum* has a positive relationship with electrical conductivity (EC), pH and OC of the surface layer (0–30 cm), and a negative relationship with EC and OC of the subsurface layer (30–60 cm). According to the specified optimal threshold (0.11), the degree of accommodation of the forecast map with the real map is at a medium level (0.5) (Table 5).

Soil parameters are also identified as the most effective factors in the distribution of this species in the Mehriz habitat (Eq. 6). The presence of *D. ammoniacum* has a positive relationship with K, HCO_3_ and SO_4_ of the surface layer (0–30 cm), and a negative relationship with Silt, Clay and SO_4_ of the subsurface layer. According to the specified optimal threshold (0.35), the degree of accommodation of the forecast map with the real map is at a good level (0.67) (Table 5). The modeled presence and absence area of *D. ammoniacum* is shown in Fig. 3.

4 $$P\left(D.am\right)Nadoushan=\frac{{\text{exp}}\left(\text{0.29Sp1)+(0.073Na1)+(18.95OC2)+(-185.95N1)+(0.42Ca1)}-11.29\right)}{1+{\text{exp}}\left(\text{0.29Sp1)+(0.07Na1)+(18.95OC2)+(-185.95N1)+(0.42Ca1)}-11.29\right)}$$

5 $$P\left(D.am\right)Bafgh=\frac{{\text{exp}}\left(\text{0.52EC1-0.73EC2+78.034pH1+620.25OC1-894.94OC2-356.35}\right)}{\text{1+exp}\left(\text{0.52EC1-0.73EC2+78.03pH1+620.25OC1-894.94OC2-356.35}\right)}$$

6 $$P\left(D.am\right)Mehriz=\frac{{\text{exp}}\left(8.01K1+2.28Hco31+0.90So41-1.06Silt1-1.14Clay1-0.46So42+0.45\right)}{\text{1+exp}\left(8.01K1+2.28Hco31+0.90So41-1.06Silt1-1.14Clay1-0.46So42+0.45\right)}.$$

### Maximum entropy

**Figure 2:**
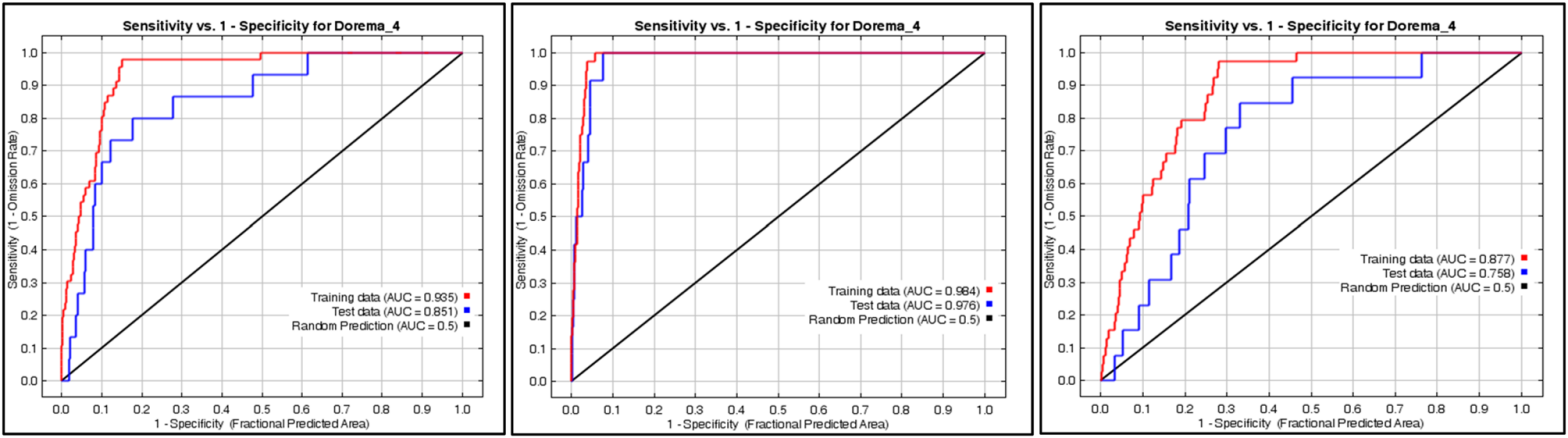
ROC curves of sensitivity vs. specificity (Training data: 75%, Test data: 25%). (From left to right, Bafagh, Nadoushan and Mehri habitats, respectively).

The distribution map of species *D. ammoniacum* obtained using the MaxEnt method is presented in Fig. 2. Figure 2 illustrates the area under the receiver operating characteristic (ROC) curve, commonly referred to as AUC. These AUC values facilitate straightforward comparisons of model performance and are instrumental in evaluating various MaxEnt models^15^. The accuracy of the prediction model based on the classification is at an acceptable level for Bafgh habitat (AUC = 0.76) and the Nadoushan habitat (AUC = 0.85). For the Mehriz habitat, the accuracy of prediction is excellent (AUC = 0.98).

**Figure 3:**
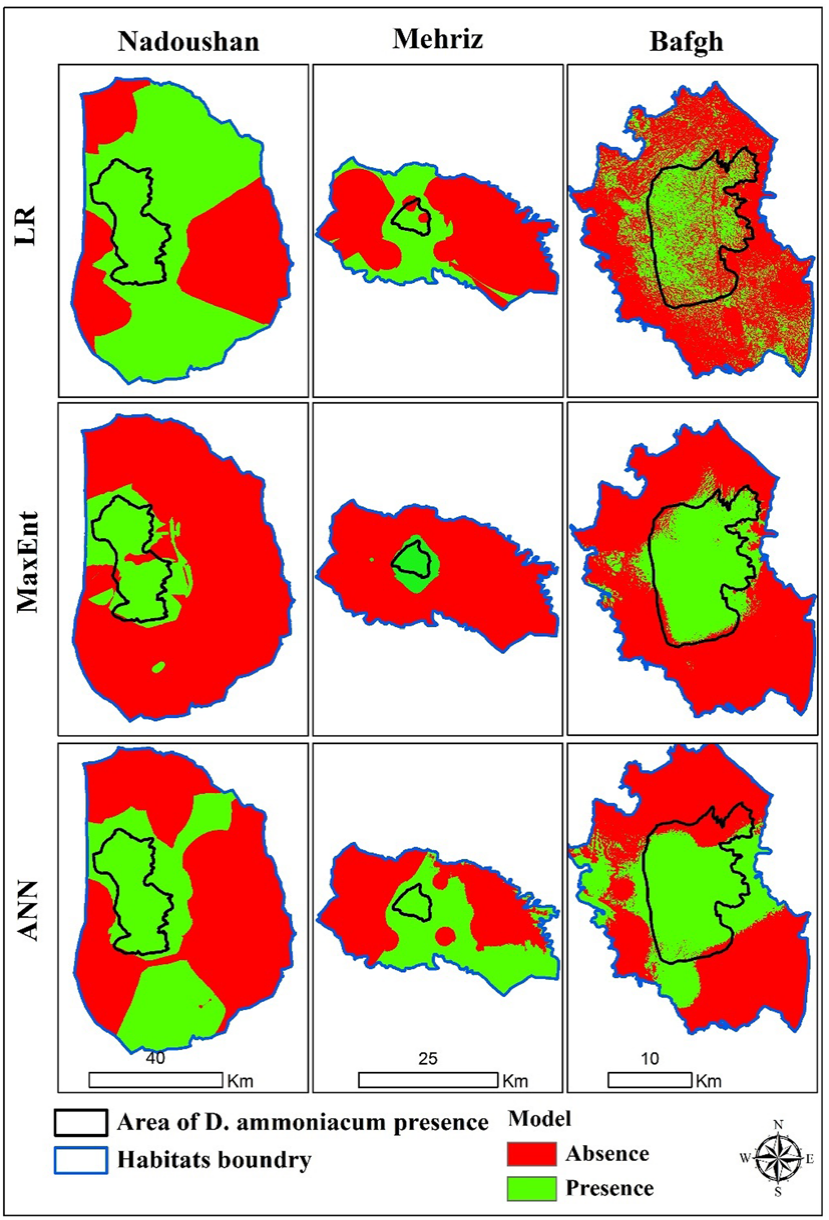
Modeled maps of D. ammoniacum species using three methods in three habitats (The processing and creation of spatial change maps resulting from species distribution modeling were conducted by the researchers using ArcGIS version 10.1^39^).

In Bafgh, the results of the Jackknife method showed that the factors of OM, OC, and N of the surface layer and EC and sand of the subsurface layer are the most important and effective factors. At Mehriz habitat, Na of subsurface layer, Ca, and Silt of surface layer, and slope have a significant relationship with the distribution of *D. ammoniacum*. At the Nadoushan habitat, the most important variables with the largest share in the model are: N, OC, OM of surface layer, and N, OM of subsurface layer. The results of the accuracy assessment of the models (Kappa coefficient) are shown in Table 5. The final prediction maps were based on two levels of presence (1) and absence (0) of plant species (Fig. 3).

### Artificial neural network

Various parameters of the ANN, including the momentum training module, the axon transfer tangent function, and the number of hidden layer neurons and processing elements, were tested to design an appropriate network. The trial and error method was employed to refine the neural network model^83^. Th e results of the potential distribution of *D. ammoniacum* in the studied habitats by ANN indicate that this species may be present in wider areas. Table 3 shows the results of the implemented models for each habitat. The model was executed using a sigmoid function with 10,000 replications. Accuracy rate was calculated for Bafgh and Nadoushan habitats as 96.43% and 93.33% and for Mehriz habitat as 100%. The forecast map model was prepared after training (Fig. 3).

**Table 3:** Executed model parameters.

Information from multilayer perceptron (MLP)			
Parameters/habitats	Bafgh	Mehriz	Nadoushan
Output function	Sigmoid	Sigmoid	Sigmoid
RMSE, acceptable	0.01	0.01	0.01
Iterations	10,000	10,000	10,000
Training RMS	0.24	0.10	0.24
Testing RMS	0.23	0.15	0.28
Accuracy rate (%)	96.43	100	93.33
Skill measure	0.93	1.00	0.87

Based on the results of the ANN, the most important variables affecting the distribution of species *D. ammoniacum* in Bafgh habitat are altitude, N of surface layer, and EC, N, OC, pH of subsurface layer. In Mehriz habitat, the most important variables affecting the distribution of the studied species are pH of the subsurface layer, Ca, N, SO_4_ of the surface layer, temperature, altitude, precipitation, and slope. In the Nadoushan habitat, the most important variables affecting the distribution of the studied species are Clay, K, N of surface layer, and N of the subsurface layer (Table 4).

**Table 4:** Range of changes the most important influential variables of species *D. ammoniacum* in the studied habitats.

Habitats	Modeling methods	Influential variables	Variation range	Unit
Bafgh	RL	EC (0–30 cm)	0.5–2	dS/m
		pH (0–30 cm)	5.18–8.05	–
		OM (0–30 cm)	0.7–1	%
	MaxEnt	OM (0–30 cm)	0.7–1	%
		OC (0–30 cm)	0.45–0.85	%
		N (0–30 cm)	0.04–0.07	%
		EC (30–60 cm)	0.5–2	dS/m
	ANN	altitude	1887–2215	m
		N (0–30 cm)	0.02-0.03	%
		EC (30–60 cm)	0.39–0.62	dS/m
		N (30–60 cm)	0.034–0.035	%
		OC (30–60 cm)	0.13–0.32	%
		pH (30–60 cm)	7.15–8.05	–
Mehriz	LR	K (0–30 cm)	0.64–1.96	ppm
		HCO_3_ (0–30 cm)	2.60–5.50	meq/l
		SO_4_ (0–30 cm)	7.80–72.50	meq/l
	MaxEnt	Na (30–60 cm)	1–5.5	ppm
		Ca (0–30 cm)	4–6	ppm
		Silt (0–3 cm)	14–18	%
		Slope	1–2.5	%
	ANN	pH (30–60 cm)	8.10–9.55	–
		N (30–60 cm)	0.04–0.058	%
		Ca (0–30 cm)	4–38.67	meq/l
		SO_4_ (0–30 cm)	7.8–72.50	meq/l
		temperature	19.26	°C
		altitude	1937–2094	m
		precipitation	67.31	mm
		slope	1–2.5	%
Nadoushan	LR	SP (0–30 cm)	22–38	%
		Ca (0–30 cm)	4.5–7	meq/l
		Na (30–60 cm)	1.3–6.0	meq/l
		OC (30–60 cm)	0.4–0.78	%
	MaxEnt	N (0–30 cm)	0.05–0.07	%
		OC (0–30 cm)	0.4–0.78	%
		OM (0–30 cm)	0.5–0.9	%
		N (30–60 cm)	0.04–0.055	%
		OM (30–60 cm)	0.2–0.4	%
	ANN	Clay (0–30 cm)	14–18	%
		K (0–30 cm)	12–16	ppm
		N (0–30 cm)	0.05–0.07	%
		N (30–60 cm)	0.04–0.055	%

In general, the range of changes of the most important variables affecting the distribution of species *D. ammoniacum* across all three habitats and based on all three models used, are detailed in Table 4. The environmental factors that consistently impact species distribution within each habitat are highlighted in bold within the table. The Maxen, Maxent, and ANN models emerged as the most accurate predictors for the Bafeq, Mehriz, and Nadoushan habitats, respectively, as highlighted in Table 4.

The degree of agreement of the prediction maps obtained from the neural network model with the terrestrial reality in the studied habitats of *D. ammoniacum* is presented in Table 5. Based on the results obtained from the evaluation of the models with Kappa coefficient, the degree of agreement of the predicted map with the terrestrial reality related to Bafgh habitat was excellent. The rate of adaptation was good for Mehriz habitat and excellent for Nadoushan habitat.

**Table 5:** Optimal presence threshold and degree of accordance of *D. ammoniacum* species prediction map with terrestrial reality based on three mode.

Model	Habitat	Threshold	Kappa coefficient	Agree between real map vs prediction map
LR	Bafgh	0.11	0.50	Medium
	Mehriz	0.35	0.67	Good
	Nadoushan	0.21	0.56	Good
MaxEnt	Bafgh	0.37	0.89	Excellent
	Mehriz	0.15	0.89	Excellent
	Nadoushan	0.28	0.78	Very good
ANN	Bafgh	0.53	0.87	Excellent
	Mehriz	0.50	0.63	Good
	Nadoushan	0.45	0.90	Excellent

The LR, MaxEnt, and ANN models were used to determine the areas classified as suitable and unsuitable for *D. ammoniacum* within each habitat, as shown in Table 6. The results of the modeling framework offer valuable insights into the potential presence of *D. ammoniacum* in the study area, facilitating the implementation of management strategies for its effective long-term conservation.

**Table 6:** Level of suitable/unsuitable area using LR, MaxEnt and ANN models based on thresholds in modeling habitat suitability of *Dorema ammoniacum* D Don.

Habitat	Total area (Km^2^)	Current habitat area (Km^2^)	Habitat suitability	Area (Km^2^)		
				LR	MaxEnt	ANN
Bafgh	645	172	Suitable	204	202	260
			Unsuitable	441	443	385
Mehriz	739	26	Suitable	211	61	334
			Unsuitable	528	678	405
Nadoushan	4557	500	Suitable	2882	3177	1783
			Unsuitable	1675	1380	2774

## Discussion

Endemic and rare species have acquired top preference for conservation because of their extinction risk. We used three methods of LR, MaxEnt, and ANN for conservation of *D. ammoniacum*, a threatened and endemic species of Yazd province. An assessment of the model efficiency in this study showed that the accuracy of the models is different in predicting the presence or absence of species studied. Although, it should be noted that no modeling method is able to perform best in all conditions and the selection of the optimal modeling method, in addition to statistical considerations, is a function of factors such as the purpose of modeling, the ecological characteristics of the species, the type of data available and the interpretability of the results from an ecological point of view^22^. In general, modeling, simulating, and mapping potential habitats for these species can aid in conservation planning and management. Salam et al.^1^ reported similar results. In this study, the modeled maps showed good predictive accuracy (Kappa coefficient = good, very good, and excellent), and the environmental factors were identified as important determinants of the *D. ammoniacum* distribution. According to the Kappa coefficient, the best and most accurate model in predicting species distribution in the study area was MaxEnt, then ANN and LR. Therefore, MaxEnt model can be considered as an effective tool for the conservation of D. ammoniacum habitat in this area. This result corresponded with Abdelaal^28^; Esfanjani et al.^32^ and Yan et al.^31^ studies.

In Bafgh habitat, the accuracy of the predicted maps using the MaxEnt method was higher than the other methods. The results of the Jackknife operation in this study indicated that soil properties are the most effective parameters in the distribution of species D. ammoniacum. OM, OC, and N of the surface layer (0–30 cm), and EC and sand percentage of subsurface layer (30–60 cm) had the greatest effect in predicting the presence of the species. The probability of the presence of species *D. ammoniacum* in Bafgh habitat is directly related to the increase in the amount of OC, OM, and soil N, so that it can be said that with increasing the amount, the conditions for settling this species are suitable introduced top OC, OM and soil N. Organic matter is very low in soils of arid and semi-arid regions, but has many effects on soil physical, chemical and biological properties. Therefore, OM is a limiting substance in these areas. Also, in the natural environment, OM plays a critical role in sourcing nutrients like N, P, and K for plants^17^. The effect of soil OC and OM on the distribution and presence of plant species has been confirmed in studies by other researchers^68,82^. In a study of the prediction of plant *D. ammoniacum* density in degraded rangelands south of Sabzevar (Iran), the correlation coefficient between the plant distribution and organic matter was 0.85^84^. Soil OM and OC are the most important factors affecting species diversity and should be considered in managing the promotion of species diversity^85^. Additionally, in this study, soil N was also identified as one of the effective factors on the distribution of the studied species. One of the reasons for this can be that N is a very effective element in the expansion and presence of plant species and has the highest amount of element absorption in plants. In other words, the effectiveness of these elements is due to their high mobility^86^. Also, N, OM, and OC are indicators of soil quality (Jannat Babaei et al. 2019). Habitat requirements of D. ammoniacum indicated that the highest presence probability of this species occurred in soil with high OM (0.42–1.39%), OC (0.17–0.54%), N (0.021–0.07%) of surface layer and sand (47–84%) of subsurface layer.

In Mehriz habitat, the accuracy of modeled maps using MaxEnt method was the highest among the methods studied. The soil and physiographic properties were identified as the most effective factors in the distribution of species *D. ammoniacum*. Sodium (Na) of subsurface layer (30–60 cm), slope, Ca, and Silt of surface layer (0–30 cm) had the greatest effect in the presence of the species. The probability of the presence of species *D. ammoniacum* in this habitat is inversely related to the increase in soil salinity or sodium; And with increasing soil salinity, the probability of the presence of the studied species decreases. It can be concluded that saline soil cannot meet the ecological needs of this species. The tolerance range of the studied species is from 0 to 10.9. In soils with high salinity, due to high Na ions, poisoning may occur in the plants. Also, large amounts of NaCl in soil may affect plant growth, germination, and production^16^. As was found in this study Ghorbani et al.^30^, also reported a direct relationship between the predictions of the distribution of *Leucanthemum vulgare* Lam., with Na amount. In studying relationship between soil parameters and the distribution of species *D. ammoniacum* and *Rheum ribes* in rangelands of Baghedar region, Iran. Na was also identified as the most effective parameter^87^. Another influential factor determining the distribution of the studied species is Ca of surface soil layer. In investigating the relationship between soil, and vegetation in saline lands of Qom province (Iran), K and Ca were identified as the most influential factors in the community of *Halanthium rariflorum*^88^. Also, in order to determine the relationship between soil properties and vegetation in Baghdar rangelands in Bafgh, Iran, Ca had the greatest effect on the separation of *D. ammoniacum* and *Rheum ribes*^87^. The results showed that slope is another important factor among other variables. The probability of presence of species *D. ammoniacum* in this habitat is inversely related to the increase of slope and with increasing slope, the probability of presence of the studied species decreases so that it can be concluded that high and mountainous areas reduce the probability of presence of this species. In confirmation of this research finding, it is reported that slope is the only non-soil factor that has a moderate correlation with species composition^89^. Also, slope percentage is one of the most important factors that affect plant moisture. The effect of slope was also recognizable in the depth of the soil and thus, in plant establishment^15^. The relationship between slope percentage and plant species distribution has been emphasized in other studies^22^. The amount of silt had a significant effect on the occurrence of this species especially *D. ammoniacum*. In a specific climatic area, soil texture has a greater impact on successful plant growth and regeneration than soil chemical fertility and is one of the features in the growth and establishment of plant species^68^. Studies by other researchers have emphasized the relationship between the percentage of silt and soil texture in the distribution of plant species^17,23,89^. The habitat requirements of *D. ammoniacum* indicated that the highest presence probability of this species occurred in soil with high Na (0.82–10.9%), Ca (2.1–9.5%), and Silt (4–18%) content.

In Nadoushan habitat, the accuracy of the ANN modeling method was the highest. Based on the results of sensitivity analysis, the most important variables affecting the distribution of the studied species were clay, K and N of surface layer. In this habitat, the percentage of clay at the first soil depth is effective in dictating the distribution of this species. So that the probability of the presence of this species has an inverse relationship with the percentage of soil clay and with increasing soil clay the probability of the presence of the studied species decreases. The soil textures in this habitat are sandy, sandy-clay and sandy-loam. In fact, the relationship between clay and vegetation confirms the relationship with soil texture (percentage of clay, sand, and silt). The impact of soil texture in increasing the habitat suitability of plant species has been confirmed in other studies^14,90^. Another effective factor determining the distribution of the studied species was soil N. Nitrogen is important in soil OM and plays an effective role in soil fertility^18^. The importance of N on plant distribution has been emphasized by other researchersv^15,18,22^. Another influential variable in this study is K. Other researchers have concluded that soil nutrients are a key factor in determining plant distribution^43,90^. Studying the habitat requirements of *D. ammoniacum* indicated that the highest presence probability of this species occurred in soil with high N (0.007–0.067%), clay (6–18%), K (0.9–14.6%), and lime (12–15%) content.

## Conclusions

*D. ammoniacum* is an endemic and threatened plant species in the rangelands of Yazd province, central Iran. It is facing imminent threat due to over-grazing, fragile habitat, construction of roads and over-exploitation for local use, and requires immediate conservation. In Bafgh and Mehriz habitats, among the modeling methods used, the MaxEnt method based on Kappa coefficient had better predictive performance. The MaxEnt method had a better performance because it estimated the probability of occurrence of the studied species based on the limitations related to environmental factors and by the model of MaxEnt. Accordingly, in Bafgh habitat, the variables of OC, OM and N had the highest share in the occurrence of the studied species and in Mehriz habitat, the variables of Na, Ca and soil Silt percentage had the highest share in the presence of the selected species. Therefore, the studied species prefers soil habitats with higher OM and OC, Na percentage and consequently lower salinity, lower silt content and lighter texture. According to the available information, the northern areas of Bafgh habitat and the southern areas of Mehriz habitat are more suitable for the establishment of the studied species. In Nadoushan habitat, among the modeling methods used, the ANN method based on kappa coefficient had better predictive performance. The ANN method performed better because of its ability to recognize complex nonlinear relationships. According to this method, which has produced the most accurate results, the variables of clay percentage, K and soil N had the largest share in the occurrence of the studied species. According to the available information, the southern areas of Nadoushan habitat are more suitable for species establishment. In general, in the present study, we found that MaxEnt and ANN are the best modeling methods.

Currently, the acquisition of accurate information for protective measures and prioritization poses challenges. Documenting the distribution data of the valuable plant *D. ammoniacum* stands as a crucial step towards addressing these challenges. Predictive maps derived from modeling efforts can aid rangeland experts and managers in better prioritizing actions, such as identifying suitable regions for *D. ammoniacum* establishment and restoration, and designating conservation units for its preservation. Future studies should extend beyond assessing suitable cultivation areas to exploring changes in habitat size under different climate change scenarios, thus enabling decision-making based on real conditions. Population viability analysis can further enhance conservation efforts by providing insights for effective species preservation. Given the research priority accorded by the General Department of Natural Resources and Watershed Management of Yazd Province, Iran, to investigate and protect *D. ammoniacum* habitats, the research encountered no significant limitations due to collaborative efforts with various organizations.

## Data Availability

All data generated or analysed during this study are included in this published article.

## References

[1] Salam N, Reshi ZA, Shah MA (2020). Habitat suitability modelling for Lagotis cashmeriana (ROYLE) RUPR., a threatened species endemic to Kashmir Himalayan alpines. Geol. Ecol. Landsc..

[2] Markham J (2014). Rare species occupy uncommon niches. Sci. Rep..

[3] Myers N, Mittermeier RA, Mittermeier CG, Fonseca G, Kent J (2000). Biodiversity hotspots for conservation priorities. Nature.

[4] Silva TR, Medeiros MB, Noronha SE, Pinto JR (2017). Species distribution models of rare tree species as an evaluation tool for synergistic human impacts in the Amazon rainforest. Braz. J. Bot..

[5] Lyons K, Schwartz M (2001). Rare species loss alters ecosystem function—Invasion resistance. Ecol. Lett..

[6] Lyons KG, Brigham CA, Traut BH, Schwartz MW (2005). Rare species and ecosystem functioning. Biol. Conserv..

[7] Nazeri M, Jusoff K, Bahman AR, Madani N (2010). Modeling the potential distribution of wildlife species in the Tropics. WJZ.

[8] Polak T, Saltz D (2011). Reintroduction as an ecosystem restoration technique. Biol. Conserv..

[9] Rodríguez-Salinas P, Riosmena-Rodriguez R, Arango GH, Muñiz-Salazar R (2010). Restoration experiment of* Zostera marina L.* in a subtropical coastal lagoon. Ecol. Eng..

[10] Seddon PJ, Grrifiths CG, Soorae PS, Armstrong DP (2014). Reversing defaunation: Restoring species in a changing world. Science.

[11] Funk VA, Richardson KS (2002). Systematic data in biodiversity studies: Use it or lose it. Syst. Biol..

[12] Hijmans R, Garrett KA, Huaman Z, Zhang DP, Schreuder M, Bonierbale M (2000). Assessing the geographic representativeness of genebank collections: The case of Bolivian Wild Potatoes. Biol. Conserv..

[13] Ghorbani A, Samadi Khangah S, Moameri M, Esfanjani J (2020). Predicting the Distribution of *Leucanthemum*
*Vulgare* Lam. using logistic regression in Fandoghlou rangelands of Ardabil province, Iran. J. Rangel Sci..

[14] Hosseini SH, Heshmati GA, Mirza M, Karami P (2019). Effects of altitude gradient and physical and chemical soil factors on functional and distribution characteristics of *Ferula haussknechtii* (Case study: Bayenchob Rangelands, Saral of Kurdistan). Iran J. Range Desert Res..

[15] Esfanjani J, Ghorbani A, Zare Chahouki MA (2018). MaxEnt modeling for predicting impacts of environmental factors on the potential distribution of *Artemisia aucheri* and *Bromus tomentellus-Festuca ovina* in Iran. Pol. J. Environ. Stud..

[16] Ghorbani A, Moameri M, Dadjou F, Seyedi Kaleybar S, Pournemati A, Asghari S (2020). Determinization of environmental factors effects on plants production in QezelOzan-Kosar rangelands, Ardabil province. ECOPERSIA.

[17] Zare Chahouki MA, Abbasi M (2018). Habitat prediction model medicinal species of *Rheum*
*ribes* L. with maximum entropy model in Chahtorsh rangeland of the Yazd province. J. Range Water Manag..

[18] Piri Sahragard H, Ajorlo MA (2018). Comparison of logistic regression and maximum entropy for distribution modeling of range plant species (a case study in rangelands of western Taftan, southeastern Iran). Turk. J. Bot..

[19] Carter GM, Stolen ED, Breininger DR (2006). A rapid approach to modeling species-habitate relationships. J. Biol. Conserv..

[20] Ripley BD (2007). Pattern Recognition and Neural Networks.

[21] Elith J (2006). Novel methods improve prediction of species’ distributions from occurrence data. Ecography.

[22] Piri Sahragard H, Zare Chahouki MA, Ajorlo M, Nohtani M (2017). Predictive habitat distribution modeling of *Amygdalus Scoparia* Spach in Moshakieh rangelands of Qom province. JFWP.

[23] Piri Sahragard H, Zare Chahouki MA (2015). An evaluation of predictive habitat models performance of plant species in Hoze soltan rangelands of Qom province. Ecol. Model..

[24] Cao B, Bai C, Zhang L, Li G, Mao M (2016). Modeling habitat distribution of *Cornus officinalis* with Maxent modeling and fuzzy logics in China. Plant Ecol..

[25] Yılmaz H, Yilmaz O, Akyüz Y (2017). Determining the factors affecting the distribution of* Muscari latifolium*, an endemic plant of Turkey, and a mapping species distribution model. Ecol. Evol..

[26] Wei B, Wang R, Hou K, Wang X, Wu W (2018). Predicting the current and future cultivation regions of *Carthamus*
*tinctorius* L. using MaxEnt model under climate change in China. Glob. Ecol. Conserv..

[27] Ngoy KI, Shebitz D (2019). Characterizing the spatial distribution of* Eragrostis Curvula* (Weeping Lovegrass) in New Jersey (United States of America) using logistic regression. Environments.

[28] Abdelaal M, Fois M, Fenu G, Bacchetta G (2019). Using MaxEnt modeling to predict the potential distribution of the endemic plant *Rosa*
*arabica* Crép. Egypt. Ecol. Inform..

[29] Pecchi M, Marchi M, Burton V, Giannetti F, Moriondo M, Bernetti I, Bindi M, Chirici G (2019). Species distribution modelling to support forest management. A literature review. Ecol. Model..

[30] Ghorbani A, Samadi Khangah S, Moameri M, Esfanjani J (2020). Predicting the distribution of *Leucanthemum*
*Vulgare* Lam. Using logistic regression in Fandoghlou rangelands of Ardabil Province. Iran J. Range Sci..

[31] Yan H, Feng L, Zhao Y, Feng L, Wu D, Zhu C (2020). Prediction of the spatial distribution of *Alternanthera philoxeroides* in China based on ArcGIS and MaxEnt. Glob. Ecol. Conserv..

[32] Esfanjani J, Ghorbani A, Moameri M, Zare Chahouki M, Esmali Ouri A, Mirzaei Mossivand A (2020). Prediction of distribution of *Prangos*
*uloptera* DC. using two modeling techniques in southern rangelands of Ardabil province. Iran J. Range Sci..

[33] Mozaffarian V (2003). Dictionary of Iranian plant names.

[34] Ajani Y, Ajani M (2008). A new species of Ferula (Umbelliferae) from southern Iran. Edinb. J. Bot..

[35] Mozaffarian V (2007). Flora of Iran, Umbelliferae Family.

[36] Moghimi J (2005). Introduction of Some Important Rangeland Species.

[37] Amin GH (2005). Popular Medicinal Plants of Iran.

[38] Rajani M, Saxena N, Ravishankara MN, Desai N, Padh H (2002). Evaluation of the antimicrobial activity of ammoniacum gum from *Dorema ammoniacum*. Pharm. Biol..

[39] ESRI. ArcGIS Desktop: Release 10.1 Redlands, CA: Environmental Systems Research Institute (2011).

[40] Haidari M, Jalilvand H, Heidari RH, Shabaniain N (2012). Study of plant biodiversity in grazed and non-grazed areas in the Iran-o-Turanian ecological zones (case study: Yazd province, Iran). J. Biol. Res..

[41] Dadjou F, Ghorbani A, Moameri M, Bidarlord M (2018). Effects of temperature and rainfall on the aboveground net primary production of Hir and Neur rangelands in Ardabil province. Iran J. Range Desert Res..

[42] Ghorbani A, Dadjoo F, Moameri M, Bidar Lord M, Hashemi Majd K (2018). Investigating the relationships between net primary production with physiographic factors in Hir and Neur rangelands in Ardabil province. Iran J. Range.

[43] Ghorbani A, Dadjou F, Moameri M, Bidar Lord M (2019). Effective topographic and climate factors on aboveground net primary production in Hir and Neur rangelands of Ardabil province. J. Range Water Manag..

[44] Walkley A, Black IA (1934). An examination of the degtjareff method for determining soil organic matter, and a proposed modification of the chromic acid titration method. Soil Sci..

[45] Dallal, GE. Collinearity. http://www.tufts.edu/gdallal/collin.htm (2001).

[46] Arzani H (1997). Manual of Rangeland Assessment Plan in Rangelands of Iran with Various Climate Conditions.

[47] Ghafari S, Ghorbani A, Moameri M, Mostafazadeh R, Bidarlord M, Kakehmami A (2020). Floristic diversity and distribution patterns along an elevational gradient in the northern part of the Ardabil province rangelands, Iran. Moun. Res. Dev..

[48] Gee, G. W., Bauder, J. W. Particle size analysis. In: A. Klute, (eds) Methods of Soil Analysis, Part 1: Physical and Mineralogical Methods. ASA Monograph No. 9, 2nd ed., Madison, WI: American Society of Agronomy/Soil Science Society of America, 383–411 (1982).

[49] Azmoodeh A, Kavian A, Soleimani K, Vahabzadeh GH (2010). Comparing runoff and soil erosion in forest, dry farming and garden land uses soils using rainfall simulator. J. Water Soil.

[50] Sparks DL (1996). Methods of Soil Analysis. Part 3. Chemical Methods. Soil Science Society American, Inc.

[51] Bremmer, JM. & Mulvaney, C.S. Nitrogen total. 595–624. In: A. L. Page (eds) Methods of soil analysis, part 2: Chemical and microbiological properties, vol. 9. ASA Monograph, Madison, WI (1982).

[52] Walkley A, Black IA (1934). Chromic acid titration for determination of soil organic matter. Soil Sci..

[53] Boltz DF, Howel JA (1978). Colorimetric Determination of Non-Metals.

[54] Lee J, Campbell CM (1969). Atomic absorption spectrophotometric and ethylene-diaminetetraaeetrate-titration method for calcium and magnesium determinations. J. Dairy Sci..

[55] Smirnov N (1948). Table for estimating the goodness of fit of empirical distributions. Ann. Math. Stat..

[56] Anderson TW, Darling DA (1952). Asymptotic theory of certain “goodness of fit” criteria based on stochastic processes. Ann. Math. Stat..

[57] Osborne J (2019). Improving your data transformations: Applying the Box-Cox transformation. Pract. Assess. Res. Eval..

[58] Feng C, Wang H, Lu N, Tu XM (2012). Log-transformation: Applications and interpretation in biomedical research. Stat. Med..

[59] Moussa H, Abboud M (2024). Methodology of applying inverse distance weighting interpolation method in determining normal heights. Resourceedings.

[60] Zare M, Ghorbani A, Moameri M, Piri Sahragard H, Mostafazadeh R (2020). Study of habitat suitability for Dorema ammoniacum establishment in Sabz Dasht rangelands of Bafgh, Yazd Province. Arid Biome.

[61] Behi MJ, Mokhtari MH, Moradi Gh, Saremi MA (2021). Modeling vegetation distribution based on environmental variables and logistic regression method in Mullah Fahleh area of Firoozabad, Fars province. J. Plant Res. (Iran. J. Biol.).

[62] Samadi Khanghah S, Moameri M, Ghorbani A, Mostafazadeh R, Biswas A (2022). Modeling potential habitats and predicting habitat connectivity for *Leucanthemum*
*vulgare* Lam. in northwestern rangelands of Iran. Environ. Monit. Assess.

[63] Wharton D, Wright ST, Wang Y (2012). Distance-based multivariate analyses confound location and dispersion effects. J. Methods Ecol. Eval..

[64] Phillips SJ, Anderson RP, Schapire RE (2006). Maximum entropy modeling of species geographic distributions. Ecol. Model..

[65] Ghorbani A, Dadjou F, Moameri M, Biswas A (2020). Estimating aboveground net primary production (ANPP) using landsat 8-based indices: A case study from Hir-Neur rangelands. Iran Range Ecol. Manag..

[66] Phillips SJ, Anderson RP, Schapire RE (2006). Maximum entropy modeling of species geographic distributions. Ecol. Model..

[67] Baldwin RA (2009). Use of maximum entropy modeling in wildlife research. Entropy.

[68] Hosseini SZ, Kappas M, Zare Chahouki MA, Gerold G, Erasmi S, Rafiei Emam A (2013). Modelling potential habitats for *Artemisia sieberi* and *Artemisia aucheri* in Poshtkouh area, central Iran using the maximum entropy model and geostatistics. Ecol. Inform..

[69] Mirzaeizadeh V, Mahdavi A, Naji H, Ahmadi H (2023). Modeling the distribution of species Pistacia atlantica in Ilam Province using MaxEnt methods. Ecol. Iran. Forests.

[70] Elith J, Phillips SJ, Hastie T, Dudík M, Chee YE, Yates CJ (2011). A statistical explanation of MaxEnt for ecologists. Divers. Distrib..

[71] Radha KO, Khwarahm NR (2022). An integrated approach to map the impact of climate change on the distributions of Crataegus azarolus and Crataegus monogyna in Kurdistan Region, Iraq. Sustainability.

[72] Richards DR, Friess DA (2015). A rapid indicator of cultural ecosystem service usage at a fine spatial scale: content analysis of social media photographs. Ecol. Indic..

[73] Coppes J, Braunisch V (2013). Managing visitors in nature areas: Where do they leave the trails? A spatial model. Wildl. Biol..

[74] Guisan A, Zimmermann NE (2000). Predictive habitat distribution models in ecology. Ecol. Model..

[75] Merow C, Smith MJ, Silander JA (2013). A practical guide to MaxEnt for modeling species’ distributions: What it does, and why inputs and settings matter. Ecography.

[76] Jackson RB, Schlesinger WH (2004). Curbing the US carbon deficit. Proc. Natl. Acad. Sci. U. S. A..

[77] Perry L, Chapman J (1975). Effects of clipping on dry matter yields of Basin wildrye. J. Range Manag..

[78] Hutchings MJ, John EA, de Kroon H, Visser EJW (2003). Distribution of roots in soil, and root foraging activity. Root Ecology.

[79] Zare Chahuoki MA, Abbasi M, Azarnivand H (2014). Evaluating the ability of artificial neural network model in predicting the spatial distribution of plant species (case study: Rangeland of Taleghan miany). J. Range.

[80] Karimi P, Kamangar M, Hosseini M (2016). Modelling of habitat suitability of persian Gazella (*Gazella Subgutturosa Subgutturosa*) in Qaraviz no hunting area and Kermanshah province by using artificial neural networks. J. Anim. Res..

[81] Abbasi M, Zare Chahouki MA (2014). Modeling of potential habitat for *Stipa barbata* and *Agropyron intermedium* species using artificial neural network model in rangeland of central Taleghan. Renew. Nat. Res..

[82] Bagheri H, Ghorbani A, Zare Chahouki MA, Sefidi K (2017). Halophyte species distribution modeling with MaxEnt model in the surrounding rangelands of Meighan playa, Iran. Appl. Ecol. Environ. Res..

[83] Esfanjani J, Ghorbani A, Moameri M, Zare Chahouki MA, Esmali Ouri A, Ghasemi ZS (2021). Application of modeling techniques for the identification the relationship between environmental factors and plant species in rangelands of Iran. Ecol. Inform..

[84] Ghasemi Arian A, Rezvani Moghaddam P, Naseripour Yazd M, Mesdaghi M, Ghorbani R (2017). Prediction of* Dorema ammoniacum* density in degraded rangelands with using neural network. Iran J. Biol..

[85] Sadeghpour A, Moetamedi J, Karkaj E (2019). Recognition the most important factors of physiography, topography and soil on plant diversity (case study: Namin mountain rangelands, Ardebil). Iran J. Range Desert Res..

[86] Jannat Babaei M, Moradi G, Feghhei J (2019). The effect of environmental factors on the distribution of ecological habitat groups Paliurus spina-christi Mill. (Case study: Chalus MarzanAbad). PEC.

[87] Rezai Poorbaghedar A, Sedghinia M, Nouhagar A, Hakimi MH (2014). Determination of some soil properties on distribution of vegetation types and Dorema ammoniacum and Rheum ribes in ranges of Baghedar region in Bafgh city. DEEJ.

[88] Asrari A, Bakhshikhaniki G, Rahmatizadeh A (2012). Assessment of relationship between vegetation and salt soil in Qom province. Iran J. Range Desert Res..

[89] Kargar M, Jafarian Z, Tamartash R, Alavi S (2018). Comparison of non-parametric and parametric species distribution models (SDM) in determining the habitat of dominant rangeland species (case study: Khetteh Riz Rangelands). Iran J. Range Desert Res..

[90] Zhao W, Li J, Qi J (2007). Changes in vegetation diversity and structure in response to heavy grazing pressure in the northern Tianshan Mountains, China. J. Arid Environ..

